# Preparation and Oil Adsorption of Cellulose-*graft*-poly(butyl acrylate-*N*,*N*′-methylene Bisacrylamide)

**DOI:** 10.3390/ma17020325

**Published:** 2024-01-09

**Authors:** Peng-Xiang Guo, Xin-Gang Wang, Mao-Qian Yang, Jian-Xin Wang, Fan-Jun Meng

**Affiliations:** Marine College, Shandong University, Weihai 264200, China; g326953192@163.com (P.-X.G.); yangmaoqian@126.com (M.-Q.Y.); 19906409737@163.com (J.-X.W.)

**Keywords:** cellulose, butyl acrylate, *N*,*N*′-methylenebisacrylamide, graft polymerization, oil adsorption

## Abstract

With the advancement of industrial economies, incidents involving spills of petroleum products have become increasingly frequent. The resulting pollutants pose significant threats to air, water, soil, plant and animal survival, as well as human health. In this study, microcrystalline cellulose served as the matrix and benzoyl peroxide (BPO) as the initiator, while butyl acrylate (BA) and *N*,*N*′-methylene bisacrylamide (MBA) were employed as graft monomers. Through free radical graft polymerization, cellulose-graft-poly(butyl acrylate-*N*,*N*′-methylene bisacrylamide) [Cell-g-P(BA-MBA)], possessing oil-adsorbing properties, was synthesized. The chemical structure, elemental composition, surface morphology and wetting properties of the graft polymerization products have been characterized, using infrared spectroscopy, elemental analysis, scanning electron microscopy and contact angle testing. The adsorption properties of Cell-g-P(BA-MBA) for various organic solvents and oils were then assessed. The experimental results demonstrated that Cell-g-P(BA-MBA) exhibited a maximum adsorption capacity of 37.55 g/g for trichloromethane. Adsorption kinetics experiments indicated a spontaneous and exothermic process involving physical adsorption, conforming to the Freundlich isotherm model. Furthermore, adsorption kinetics experiments revealed that Cell-g-P(BA-MBA) displayed favorable reuse and regeneration performance, maintaining its adsorption capacity essentially unchanged over fifteen adsorption–desorption cycles.

## 1. Introduction

With rapid development of modern society and economic systems, demand for various petroleum products and organic solvents has surged significantly. However, in the exploration, transportation, storage, processing, refining and utilization of petroleum, leakage accidents frequently occur, resulting in substantial waste of fossil resources and simultaneously causing severe ecological and environmental problems [1,2]. Notable tragedies include the Gulf of Mexico oil spill in 2010 and the MV Wakashio (Mauritius) oil spill in 2020 [3,4,5]. Therefore, there is a pressing need to promptly and effectively manage oil pollution to minimize economic losses and mitigate harm to ecological environments and human health.

Currently, treatment methods for oil pollution can be broadly categorized into physical, in situ combustion, chemical and biological methods [6,7]. Among these, physical methods employing oil-absorbing materials prove to be one of the swiftest approaches for removing oil spills from water surfaces [8]. In comparison with chemical and in situ combustion methods, oil-adsorbing materials have minimal or no negative impact on the environment [9,10,11,12]. These materials can be classified into three main groups: natural inorganic materials, natural organic materials and synthetic organic materials [13]. Variations in lipophilicity, mechanical strength, oil-adsorption capacity, oil retention properties and recyclability exist among these materials. Polymers, such as polyurethane, polypropylene and polyacrylate, are widely utilized as oil-adsorbing materials [2,14,15].

Ideal oil-adsorbent materials should possess such properties as high oil adsorption, high oil retention, low water adsorption and reusability, in addition to being low-cost, abundant, biodegradable and mechanically strong [16]. Considering these criteria, biopolymer materials derived from agricultural and industrial wastes are gaining increased attention [17,18].

Cellulose, widely available and inexpensive, exhibits good biodegradability, biocompatibility and mechanical properties [19,20]. M. D. Teli et al. successfully prepared natural banana fiber oil-adsorbing materials using potassium persulfate as an initiator, achieving the highest adsorption capacity for 14.45 g/g of crude oil at a monomer ratio of 1.5:1 [21]. Yang et al. synthesized RSMs-g-P(MMA-co-BA), a modified oil-adsorbing material derived from rapeseed hectoliter, using free radical graft polymerization. This modification improved mechanical and oil-adsorption properties of the polymer, maintaining an 83.42% adsorption capacity after eight repeated oil adsorptions [22]. Another study by Rong et al. involved surface activation and vinyltriethoxysilane modification of carbonized pollen particles, resulting in a three-dimensional composite resin material with an adsorption capacity of 58.8 g/g for carbon tetrachloride [23]. S Vigu et al. enhanced the oil-adsorption capacity of nettle fibers through butyl acrylate graft polymerization, achieving maximum adsorption capacities of 36.60 g/g for crude oil and 25.56 g/g for vegetable oil, surpassing those of commercial polypropylene materials [24].

In this study, a novel cellulose-based oil-adsorbing material, Cell-g-P(BA-MBA), was prepared through free radical graft polymerization using microcrystalline cellulose as a substrate, demonstrating excellent oil–water separation performance. The successful preparation of Cell-g-P(BA-MBA) was confirmed through infrared spectral analysis, elemental analysis, scanning electron microscopy analysis and contact angle analysis. This study has also investigated the adsorption kinetics, adsorption isotherm and adsorption thermodynamics of Cell-g-P(BA-MBA) for oil adsorption, as well as oil–water separation and regeneration performance.

## 2. Materials and Methods

### 2.1. Materials and Reagents

Microcrystalline cellulose (MCC) with a particle size of ≤25 μm (Aladdin’s reagent) served as the matrix, while butyl acrylate (BA) of analytical reagent grade, provided by Sinopharm Chemical Reagent Co., Ltd., Shanghai, China. was utilized as the monomer. Initiator benzoyl peroxide (BPO) of chemically pure grade was obtained from Jiangsu Strong Functional Chemistry Co., Ltd. Changshu, China. while the cross-linking agent was *N*,*N*′-methylene bis-acrylamide (MBA) (analytical reagent grade Macklin reagent, Shanghai, China). All materials and reagents were used as received without further purification.

### 2.2. Adsorbent Preparation and Characterization

**Scheme 1:**
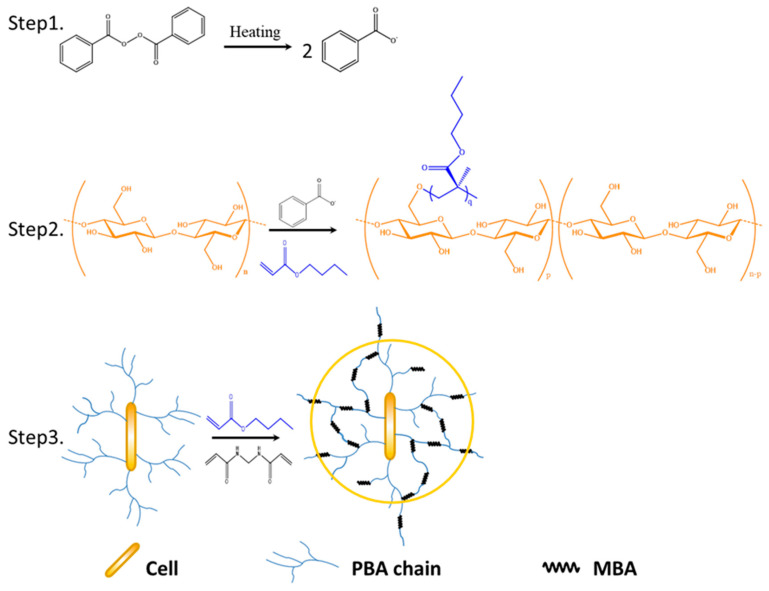
Synthetic route of Cell-g-P(BA-MBA).

1 g of Pre-soaked cellulose was introduced into a 250 mL four-necked flask and subjected to nitrogen purging for 30 min at 70 °C in a constant-temperature water bath. A prepared initiator solution of 0.09 g of benzoyl peroxide (BPO) was weighed. Under nitrogen protection, stirring commenced and the pre-prepared BPO solution was slowly added over 20 min. Subsequent to a 30 min addition of the initiator solution, 7 g of butyl acrylate (BA) monomer was added dropwise within 10 min. Following the addition of BA monomer for 30 min, a reactive monomer solution containing MBA and the remaining BA was added dropwise. The MBA dosage was 0.35 wt% of the BA monomer dosage, and the dropwise addition of the monomer solution lasted for 30 min. Upon completion of the reaction, the crude product underwent filtration and multiple washes with acetone and deionized water to eliminate unreacted initiator and monomer. Soxhlet extraction with acetone as the solvent was then conducted for 24 h to remove homopolymer of butyl acrylate. The resulting product was subjected to a vacuum drying oven at 60 °C until a constant weight was achieved. The dried product was then weighed. The reaction principle is illustrated in Scheme 1.

The preparation of Cell-g-PBA resin followed the same procedure as described above, excluding the addition of MBA during the process. Similarly, the preparation of P(BA-MBA) resin reflected the above process, with the exception that cellulose was omitted from the preparation steps.

Structure and morphology of products were examined using an FEI Nova NanoSEM 450 field emission scanning electron microscope from FEI, Hillsboro, OR, USA, operating at an accelerating voltage of 5 kV. Sample powders were affixed and coated with gold using conductive adhesive. Infrared spectra of microcrystalline cellulose, Cell-g-PBA, P(BA-MBA) and Cell-g-(PBA-MBA) were obtained with a VERTEX70 infrared spectrometer from Bruker, Germany, within a scanning range of 4000–400 cm^−1^. Prior to testing, samples were subjected to a 12 h drying period in a vacuum drying oven. Elemental analysis experiments determined the carbon (C), hydrogen (H) and nitrogen (N) content of microcrystalline cellulose, Cell-g-PBA, Cell-g-(PBA-MBA) and P(BA-MBA). The Vario EL elemental analyzer from Elementar, Germany, employing helium as purge and carrier gas, served as the testing instrument. The water contact angle of samples was measured using a DSA25 contact angle meter from Kruss, Hamburg, Germany. This test, conducted at room temperature, involved the application of 5 μL water droplets on a dry, flat sample surface whenever possible.

### 2.3. Adsorption Capacity of Oils and Organic Solvents

Adsorption capacity of Cell-g-P(BA-MBA) for 10 different oils and organic solvents was determined using the weighing method. Initially, dry oil-adsorbing material with mass *m*_1_ (approximately 0.1 g) was weighed and enclosed in a non-woven bag. Subsequently, it was fully immersed in 150 mL of respective oils or organic solvents at room temperature. After 12 h of oil adsorption, the material was promptly removed and drained for 1 min until no more droplets fell. Residual oils on the sample surface were wiped off using filter paper, and the mass of the sample (*m*_2_) was immediately recorded. This approach minimized the impact of oil and solvent volatilization on the experimental results. The adsorption capacity (*Q*) of Cell-g-P(BA-MBA) for various oils and organic solvents was calculated using Equation (1):

(1) $$\;Q=\frac{m_{2}-m_{1}}{m_{1}}$$

where *Q* (g/g) denotes the adsorption capacity per unit mass of dry oil-adsorbing material and *m*_1_ (g) and *m*_2_ (g) denote the mass of oil-adsorbing material before and after adsorption, respectively.

A control experiment using microcrystalline cellulose as the oil-adsorbing material was conducted under identical conditions.

Adsorption experiments of Cell-g-P(BA-MBA) and microcrystalline cellulose on different oils and organic solvents were repeated three times under consistent experimental conditions, with the final average value considered as the experimental result.

### 2.4. Oil–Water Separation Performance

To demonstrate the practical application of Cell-g-P(BA-MBA) in oil contamination treatment, 5 mL of Sudan III-stained toluene was dropwise added to a beaker containing 100 mL of water. Subsequently, 0.3 g of Cell-g-P(BA-MBA) was weighed and immersed into the oil–water mixture. The observation, photography and documentation were conducted at two-minute intervals.

### 2.5. Regeneration Performance

The solvent elution method was employed for regenerating Cell-g-P(BA-MBA), using hexane as the extractant [25]. The specific experimental procedure involved weighing 0.1 g of the oil-adsorbing material, placing it in a nonwoven bag and adsorbing it in 150 mL of gasoline for 12 h at room temperature. After draining and weighing to record the mass, the adsorption capacity was calculated according to Equation (1). Subsequently, the saturated oil-adsorbing material was immersed in 30 mL of hexane solution for 5 h. Following removal, draining and weighing, the mass was recorded and the adsorption capacity was calculated again using Equation (1). This procedure was repeated 15 times in gasoline and hexane to determine the adsorption capacity of Cell-g-P(BA-MBA) after each adsorption and desorption. Each set of experiments was repeated three times under the same conditions, and the final average value was considered as the experimental result.

## 3. Results and Discussion

### 3.1. Characterization of Polymers

**Figure 1:**
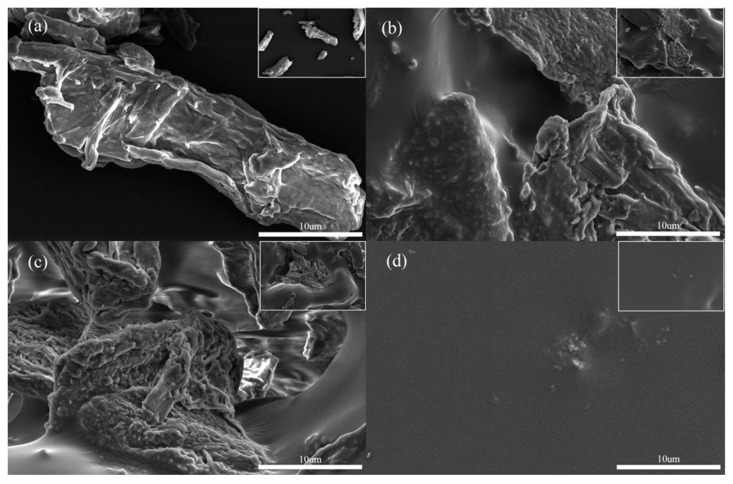
Scanning electron micrographs of (**a**) MCC, (**b**) Cell-g-PBA, (**c**) Cell-g-P(BA-MBA) and (**d**) P(BA-MBA).

The surface morphology of Microcrystalline Cellulose (MCC), Cell-g-Poly(butyl acrylate) (Cell-g-PBA), Cell-g-Poly(butyl acrylate-co-*N*,*N*′-methylenebisacrylamide) (Cell-g-P(BA-MBA)) and Poly(butyl acrylate-co-*N*,*N*′-methylenebisacrylamide) (P(BA-MBA)) was observed and analyzed using scanning electron microscopy, as depicted in Figure 1. In Figure 1a, the pristine microcrystalline cellulose appears rod-like with uneven sizes, having a longitudinal length of ≤25 μm. Upon magnification, the microcrystalline cellulose surface is observed to be nearly smooth with slight folds. Figure 1b,c reveal that polymer chains are cross-linked and entangled, accumulating on the microcrystalline cellulose surface, resulting in rougher surfaces for Cell-g-PBA and Cell-g-P(BA-MBA). Figure 1d illustrates the smooth surface morphology of P(BA-MBA). The enlarged view in Figure 1c reveals abundant folds and a small pore structure on the surface of Cell-g-P(BA-MBA), enhancing oil molecule penetration and increasing the specific surface area, thereby improving the oil adsorbent performance [26].

**Figure 2:**
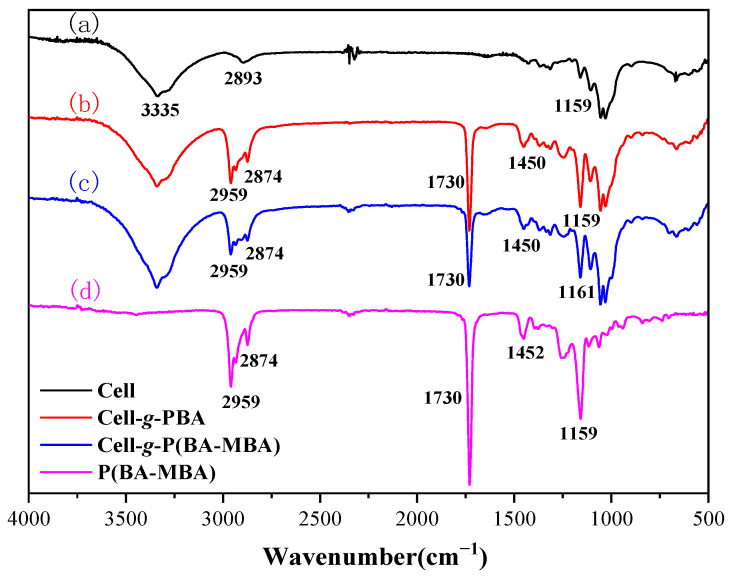
FT-IR spectra of (**a**) MCC, (**b**) Cell-g-PBA, (**c**) Cell-g-P(BA-MBA) and (**d**) P(BA-MBA).

To affirm successful preparation of Cell-g-P(BA-MBA), chemical structures of microcrystalline cellulose, Cell-g-PBA, Cell-g-P(BA-MBA) and P(BA-MBA) were characterized and comparatively analyzed using Fourier Transform Infrared (FT-IR) spectroscopy, as presented in Figure 2. In infrared spectra of microcrystalline cellulose (curve (a)), characteristic peaks include a broad adsorption band with a maximum at near 3335 cm^−1^ corresponding to the stretching vibrational adsorption of the hydroxyl H-O bond [27], a weak band with a maximum at 2893 cm^−1^ indicating the stretching vibrational adsorption of the methylene C-H bond [28] and a weak symmetric stretching vibrational adsorption band with a maximum at 1159 cm^−1^ for the C-O-C bond [29]. Cell-g-PBA and Cell-g-P(BA-MBA) exhibit new peaks, such as asymmetric and symmetric stretching vibrational adsorption peaks of methyl and methylene C-H bonds at 2959 cm^−1^ and 2874 cm^−1^ [30], a strong band with a maximum at 1730 cm^−1^ due to the stretching vibration of the C=O bond of the ester carbonyl group and an asymmetric stretching vibrational adsorption band with a maximum at 1450 cm^−1^ of the methyl C-H bond [22]. These peaks align with those in the infrared spectra of P(BA-MBA), confirming successful grafting of the P(BA-MBA) copolymer onto microcrystalline cellulose. Furthermore, the intensity of the symmetric stretching vibration adsorption peak of the C-O-C bond at 1159 cm^−1^ in curves (b) and (c) is significantly higher than that of microcrystalline cellulose, which can be attributed to the increased C-O-C content in the ester group following butyl acrylate grafting.

Elemental analysis was conducted to determine contents of C, H and N in microcrystalline cellulose, Cell-g-PBA, Cell-g-P(BA-MBA) and P(BA-MBA). The results, presented in Table 1, indicate significantly higher C and H contents in Cell-g-PBA compared to microcrystalline cellulose, affirming successful grafting of butyl acrylate onto microcrystalline cellulose. The presence of element N in Cell-g-P(BA-MBA) and P(BA-MBA) confirm successful grafting of the MBA monomer onto cellulose. Notably, the small amount of elemental N detected in the elemental analysis reflects the use of a minimal MBA monomer quantity in the preparation process to maintain optimal oil-adsorbing properties.

**Figure 3:**
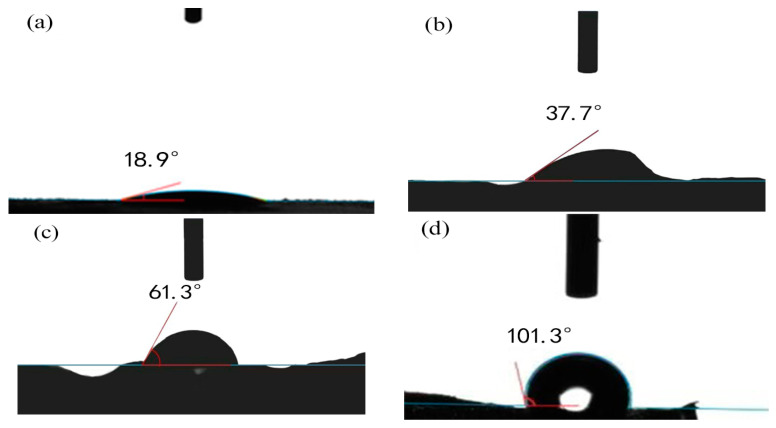
Images of water contact angles of (**a**) MCC, (**b**) P(BA-MBA), (**c**) Cell-g-PBA and (**d**) Cell-g-P(BA-MBA).

Wetting properties, crucial for oil-adsorbing material applications in oil–water separation, were assessed using water contact angle tests. Figure 3a reveals a water contact angle of 18.9° for MCC due to the hydrophilic nature of its surface hydroxyl groups. Figure 3b demonstrates that Cell-g-P(BA-MBA) attains a water contact angle of 101.3°, resulting from entangled polymer chains formed upon grafting hydrophobic BA monomers onto MCC. The presence of folds and a small pore structure on the surface of Cell-g-P(BA-MBA) enhances its hydrophobicity and lipophilicity, offering an excellent oil–water separation performance.

### 3.2. Adsorption Property Testing

**Figure 4:**
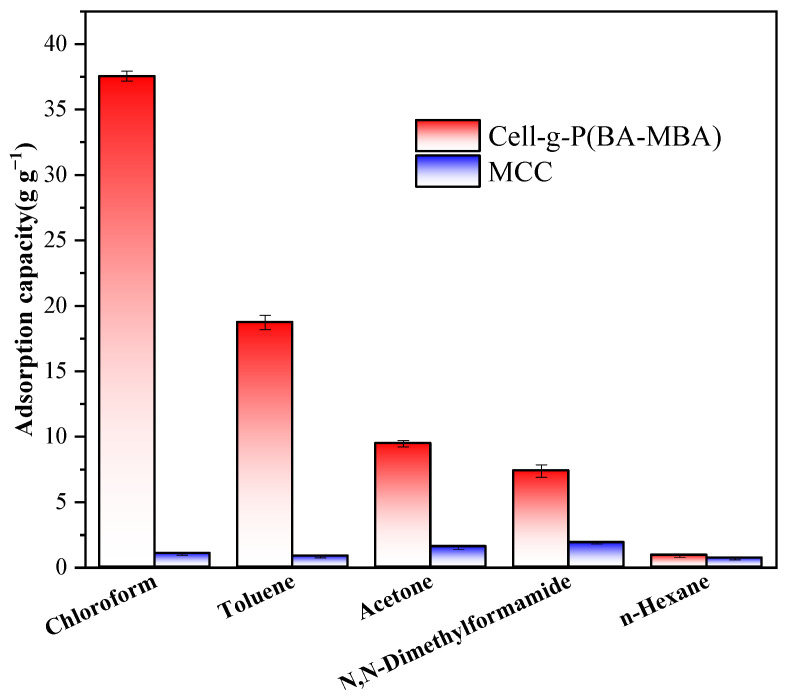
Adsorption capacity of Cell-g-P(BA-MBA) for selected organic solvents.

**Figure 5:**
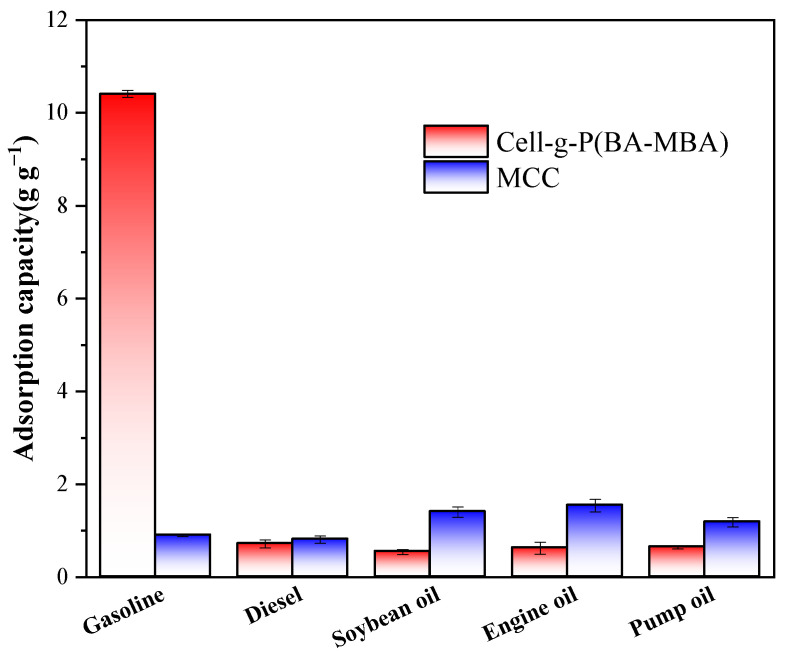
Adsorption capacity of Cell-g-P(BA-MBA) for selected oils.

Oil adsorption experiments were conducted to evaluate the adsorption capacity of Cell-g-P(BA-MBA) for various oils and organic solvents under identical conditions. The results, illustrated in Figure 4 and Figure 5, reveal a substantial influence of oil and solvent types on the adsorption capacity of Cell-g-P(BA-MBA). The material exhibits maximum average adsorption capacities of 37.55 g/g for trichloromethane, 18.72 g/g for toluene, 9.46 g/g for acetone, 7.37 g/g for *N*,*N*-dimethylformamide and 10.41 g/g for gasoline. Figure 5 shows that the maximum average adsorption capacity of Cell-g-P(BA-MBA) for n-hexane, diesel oil, soybean oil, motor oil and vacuum pump oil was in the range of 0.54 g/g–0.90 g/g. The order of adsorption capacity for different organic solvents is trichloromethane > toluene > acetone > *N*,*N*-dimethylformamide > hexane. This order correlates with the density of the selected organic solvents, suggesting that a higher density positively impacts the adsorption capacity. Additionally, the adsorption capacity of Cell-g-P(BA-MBA) for *N*,*N*-dimethylformamide, despite its lower density than acetone, is attributed to differences in polarity, where acetone interacts more strongly with Cell-g-P(BA-MBA). From Figure 5, it can be seen that the adsorption capacity of microcrystalline cellulose on diesel oil, soybean oil, motor oil and vacuum pump oil is significantly higher than that of Cell-g-P(BA-MBA), which is due to the fact that the microcrystalline cellulose used in the oil-adsorption experiments is in the form of a powder and its specific surface area is much larger than that of the Cell-g-P(BA-MBA) oil-adsorbent material, and in addition, the four kinds of oils have a larger viscosity and are therefore more likely to be adsorbed on the surface of the microcrystalline cellulose [31].

Cell-g-P(BA-MBA) demonstrates superior oil-adsorption performance compared to several other oil-adsorbing materials, as indicated in Table 2.

### 3.3. Adsorption Kinetics

**Figure 6:**
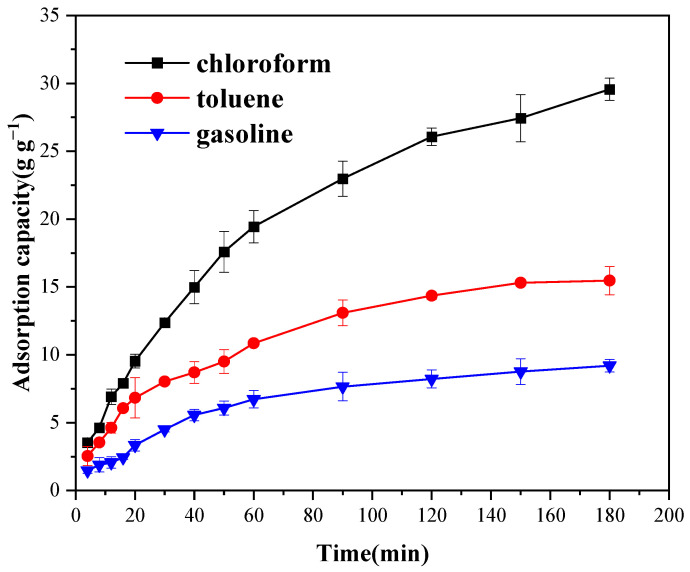
Influence of contact time on oil-adsorption capacity of Cell-g-P(BA-MBA) for oils and organic solvents within 180 min.

**Figure 7:**
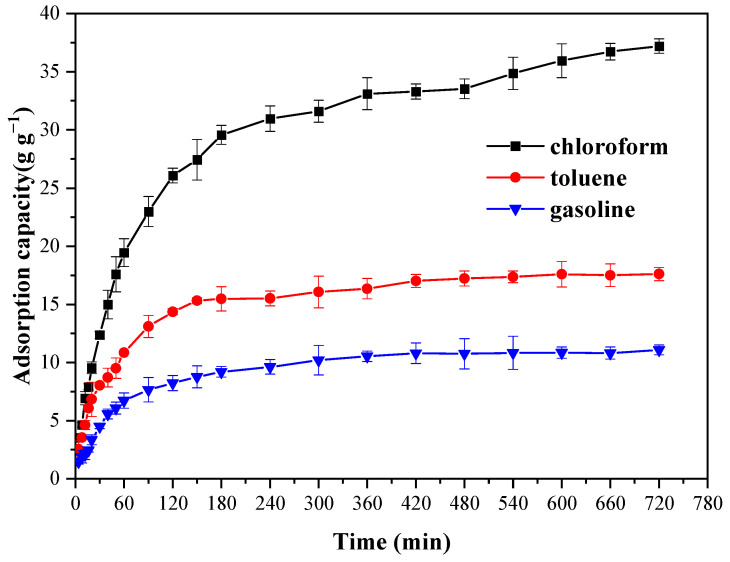
Influence of contact time on oil-adsorption capacity of Cell-g-P(BA-MBA) for oils and organic solvents within 720 min.

The oil-adsorption mechanism of Cell-g-P(BA-MBA) was systematically examined through adsorption kinetic experiments. Figure 6 and Figure 7 illustrate adsorption capacities of trichloromethane, toluene and gasoline over varying contact times. These graphs reveal a consistent pattern across three solvents, wherein adsorption rates for trichloromethane, toluene and gasoline were notably rapid within the initial 180 min. Subsequently, the rate of increase in oil-adsorption capacity diminished and the adsorption rate gradually decelerated over the subsequent 7 h. By the 10th hour of contact time, the adsorption process approached near-equilibrium levels. This stabilization persisted beyond the 10 h mark, signifying that adsorption equilibrium was nearly attained.

**Figure 8:**
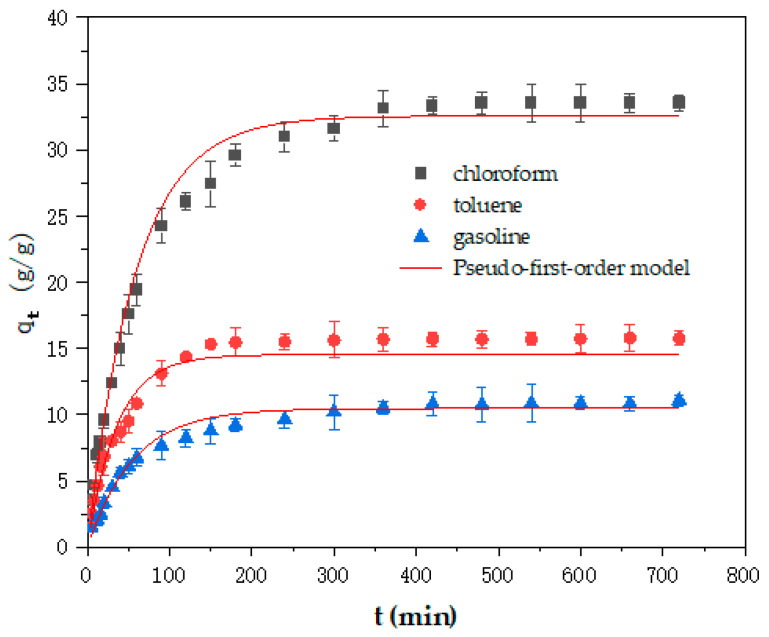
The pseudo-first-order kinetics of the Cell-g-P(BA-MBA) for the adsorption of different organic solvents and crude oil.

**Figure 9:**
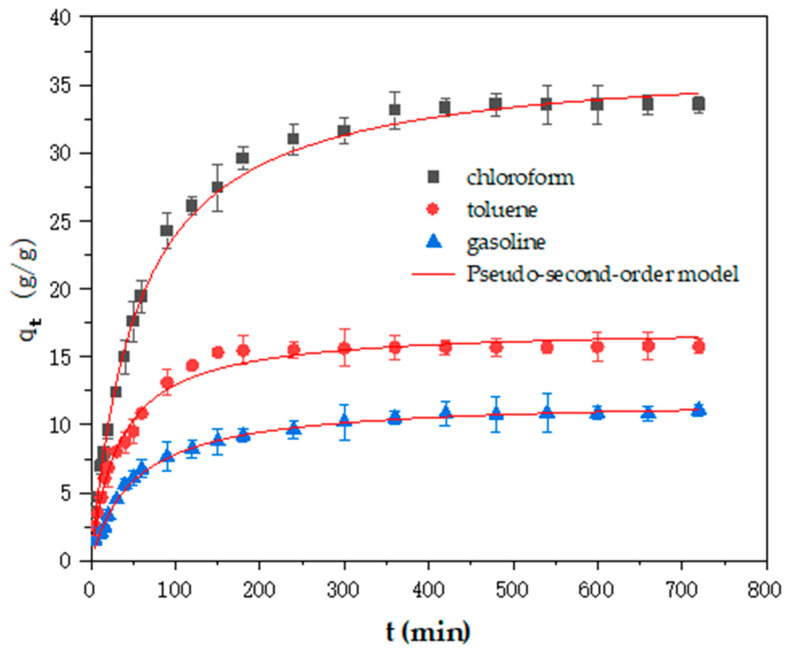
The pseudo-second-order kinetics of the Cell-g-P(BA-MBA) for the adsorption of different organic solvents and crude oil.

To elucidate the adsorption mechanism of Cell-g-P(BA-MBA) on oil and organic solvents, both a pseudo-first-order kinetic model and pseudo-second-order kinetic model were employed for non-linear fitting of the experimental data [36]. The pseudo-first-order and pseudo-second-order equation can be expressed correctly in nonlinear forms:

(2) $$q_{t}{=q}_{e}(1-e^{-k_{1}t})$$

(3) $$h=k_{2}q_{e}^{2}$$

(4) $$q_{t}=t/\left(1/h+t/q_{e}\right)$$

where q_e_ and q_t_ are the amounts of adsorbate uptake per mass of adsorbent at equilibrium and at any time t (min), respectively; k_1_ (min^−1^) and k_2_ (g/g min^−1^) are the rate constant of the pseudo-first-order and pseudo-second-order equation, respectively. The outcomes of this fitting process are depicted in Figure 8 and Figure 9. The relevant kinetic modeling parameters, including k_1_ (min^−1^), k_2_ (g/g min^−1^), q_e_, calc (g/g) and R^2^, are detailed in Table 3.

The analysis of Figure 8 and Figure 9 shows that the fitting degree of the pseudo-second-order kinetic model is significantly improved compared with the pseudo-first-order kinetic model. Upon scrutinizing the data, it becomes apparent that the correlation coefficients (R^2^ values) for the pseudo-second-order kinetic model in the adsorption process of Cell-g-P (BA-MBA) with three solvents consistently exceeded 0.98. Furthermore, the equilibrium adsorption capacities for trichloromethane, toluene and gasoline, as fitted by the pseudo-second-order kinetic model, closely reflected the actual values obtained from oil-adsorption experiments, registering at 36.98 g/g, 17.16 g/g and 11.88 g/g, respectively.

Upon comparison of the rate constants (k_2_) derived from the pseudo-second-order kinetic model, it was discerned that the adsorption rates of Cell-g-P(BA-MBA) for the three solvents followed the sequence of trichloromethane > toluene > gasoline. This order is consistent with the order of the adsorption capacity of Cell-g-P(BA-MBA) for the three solvents. The fitting results by the nonlinear method of the pseudo-second-order kinetic model are basically the same as the experimental data.

### 3.4. Adsorption Isotherm

The examination of adsorption equilibrium data through theoretical or empirical equations is crucial for understanding the interaction between adsorbent materials and adsorbates. The experimental data for gasoline adsorption were subjected to non-linear fitting using Langmuir and Freundlich isothermal adsorption models [37]. The Langmuir and Freundlich isothermal adsorption equation can be expressed correctly in nonlinear forms:

(5) $${\;q}_{e}=q_{m}K_{L}C_{e}/\left(1+K_{L}C_{e}\right)$$

(6) $$q_{e}=K_{F}C_{e}^{1/n}$$

where q_e_ is the amount of adsorbate uptake per mass of adsorbent at equilibrium, q_m_ denotes the maximum amount of sorbate, K_L_ is the thermodynamic equilibrium constant (a dimensionless quantity), C_e_ is the molar (or molal) concentration in the solution at the equilibrium and K_F_ and n are constant.

**Figure 10:**
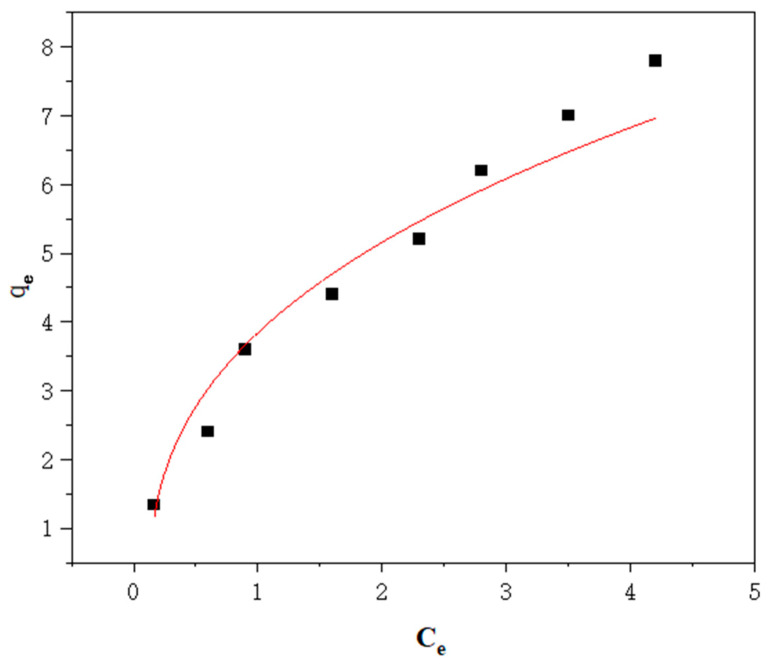
Sorption of gasoline onto Cell-g-P(BA-MBA); data fitted according to the Langmuir model.

**Figure 11:**
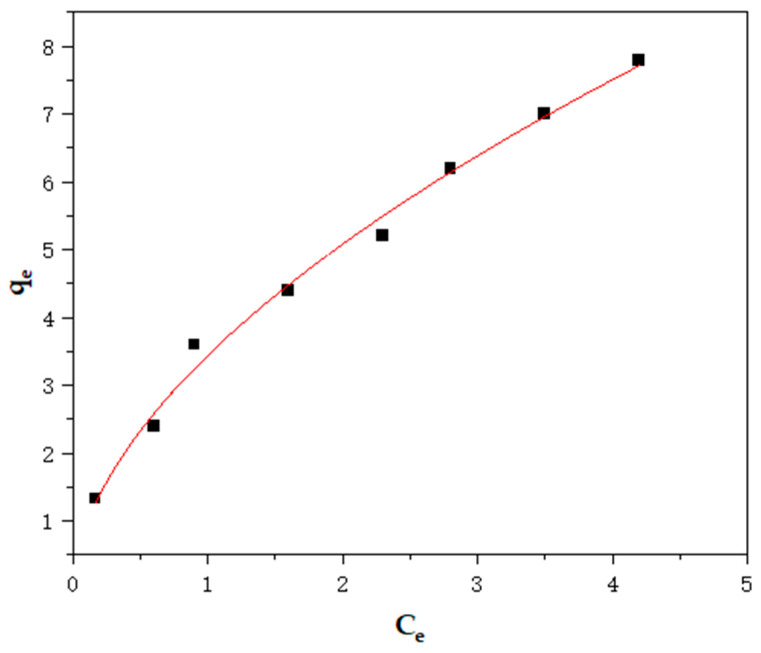
Sorption of gasoline onto Cell-g-P(BA-MBA); data fitted according to the Freundlich model.

The corresponding fitting results are presented in Figure 10 and Figure 11 and Table 4.

Comparing the correlation parameters computed by the Langmuir and Freundlich adsorption isothermal models, it was evident that the linear correlation coefficient of the Freundlich adsorption isothermal model (R^2^ = 0.99094) was significantly higher than that of the Langmuir adsorption isothermal model (R^2^ = 0.94653). This observation suggests that the Freundlich adsorption isothermal model is more suitable for describing the adsorption process of Cell-g-P(BA-MBA) oil-adsorbing materials [38]. Unlike the Langmuir model, which assumes ideal conditions of uniform adsorption in a monomolecular layer, the Cell-g-P(BA-MBA) adsorbent material’s surface is non-homogeneous, and multilayer adsorption occurs during the adsorption process. Furthermore, the n value, representing adsorption intensity, falls between 1 and 2, indicating a moderate adsorption process [39].

### 3.5. Adsorption Thermodynamics

**Figure 12:**
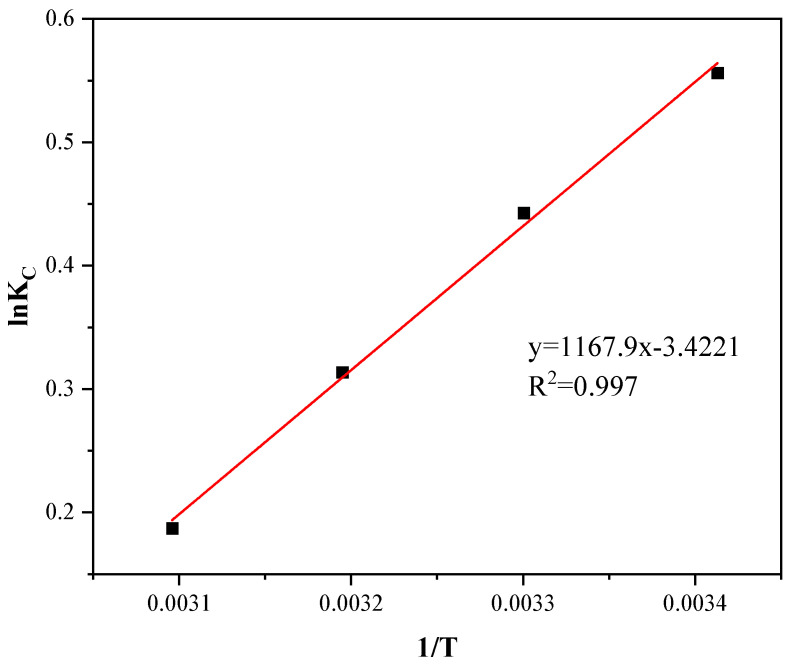
Logarithm of the equilibrium constant for the sorption of gasoline onto Cell-g-P(BA-MBA) against 1/T.

The results of fitting the experimental data on adsorption thermodynamics are depicted in Figure 12, and specific thermodynamic parameters are outlined in Table 5. As observed in Figure 12, the linear correlation coefficient R^2^ = 0.997, indicating a robust fit. From the table, it is evident that ∆G^0^ values are consistently negative across all temperatures, signifying the spontaneous nature of the adsorption process of Cell-g-P(BA-MBA) on gasoline. Absolute values of ∆G^0^ increase with decreasing temperature, suggesting that lower temperatures favor the adsorption process. According to the literature, the reported ∆G^0^ value for physical adsorption falls between −20 and 0 kJ mol^−1^, and the ∆G^0^ values obtained at different temperatures in this study are within this range, affirming the occurrence of physical adsorption during the adsorption process. Negative ∆H^0^ values indicate an exothermic adsorption process, with lower temperatures being more favorable, aligning with the previous observation. The magnitude of the ∆H^0^ value, less than 40 kJ mol^−1^, supports the notion that the adsorption process is classified as physisorption, further confirming the existence of physisorption in the adsorption process. Additionally, a negative ∆S^0^ value signifies a reduction in interfacial disorder during the adsorption process, indicative of a disorder-to-order transformation [40].

### 3.6. Oil-Water Separation Performance

**Figure 13:**
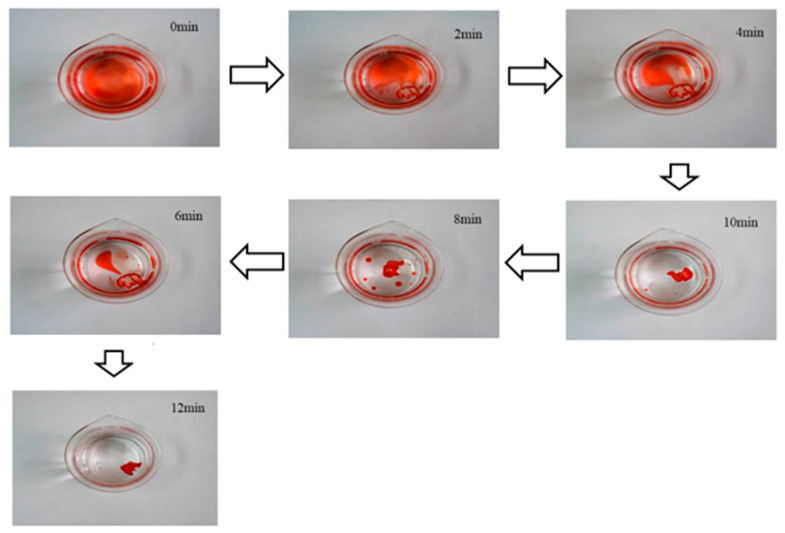
Dynamic process of toluene adsorption on water surface by Cell-g-P(BA-MBA).

The results of the oil–water separation experiments are depicted in Figure 13. Adsorption of toluene by Cell-g-P(BA-MBA) is remarkably swift; as illustrated in the figure, toluene on water surface is completely removed after approximately 12 min. Furthermore, throughout the adsorption process, the oil-adsorbing material consistently floated on the water surface, showcasing commendable buoyancy and enabling straightforward recovery. This experiment underscores that Cell-g-P(BA-MBA) exhibits notable advantages, including excellent buoyancy, a rapid oil-adsorption rate, outstanding oil–water separation performance and facile recyclability. These attributes position it as a promising candidate for deployment in emergency oil pollution treatment.

### 3.7. Regeneration Performance

**Figure 14:**
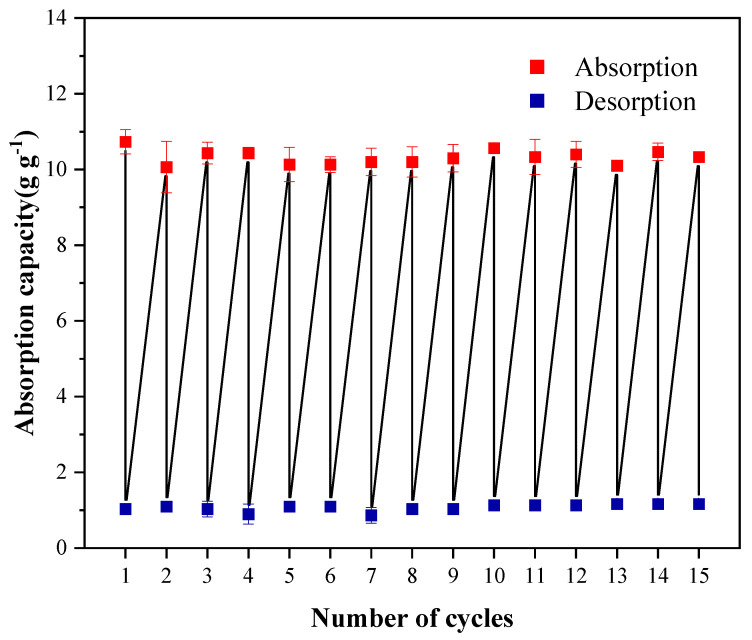
Recyclability of Cell-g-P(BA-MBA) adsorbents in gasoline.

Regeneration performance is a crucial property of oil-adsorbing materials in practical applications. In experiments, oil-adsorbing materials were reclaimed through solvent elution, and gasoline could be easily desorbed using n-hexane. The trend of the adsorption capacity of Cell-g-P(BA-MBA) on gasoline during 15 adsorption–desorption cycles is presented in Figure 14. After 15 cycles, the adsorption capacity of Cell-g-P(BA-MBA) on gasoline remained essentially unchanged compared to the initial adsorption, with no significant loss of sample mass. Cellulose in the Cell-g-P(BA-MBA) oil-adsorbing material contributed to a certain degree of mechanical strength, preserving the three-dimensional network structure. Furthermore, this experiment utilized a gentler solvent elution method for reuse, preventing damage to the structure of the Cell-g-P(BA-MBA) oil-adsorbing material in contrast to methods like drying and extrusion. These findings underscore the excellent regeneration properties of the Cell-g-P(BA-MBA) oil-adsorbing material.

## 4. Conclusions

In this study, butyl acrylate was grafted onto cellulose through free radical graft polymerization to fabricate a Cell-g-P(BA-MBA) oil-adsorbing material. The results from oil-adsorption experiments revealed that the maximum average adsorption capacities of Cell-g-P(BA-MBA) for trichloromethane, toluene, acetone, *N*,*N*-dimethylformamide and gasoline were 37.55 g/g, 18.72 g/g, 9.46 g/g, 7.37 g/g and 10.41 g/g, respectively. In addition, the maximum average adsorption capacity for n-hexane, diesel, soybean oil, engine oil and vacuum pump oil fell in the range of 0.54 g/g to 0.90 g/g. Adsorption kinetics experiments indicated that the adsorption process adhered to the quasi-secondary kinetic model, with the adsorption rate inversely correlated with the polarity of oil and organic solvent. Adsorption isotherm experiments demonstrated a non-uniform multilayer adsorption process, aligning with the Freundlich isotherm adsorption model. Thermodynamic investigations revealed the spontaneous and exothermic nature of Cell-g-P(BA-MBA) adsorption on gasoline, with lower temperatures favoring the adsorption process. The adsorption of toluene on the water surface confirmed the rapid oil treatment capability of Cell-g-P(BA-MBA). Regeneration performance experiments demonstrated that the oil-adsorption capacity of Cell-g-P(BA-MBA) remained essentially unchanged after 15 adsorption–desorption cycles. The prepared Cell-g-P(BA-MBA) exhibited notable adsorption capacities for various oils and organic solvents, coupled with economic efficiency, a straightforward preparation process and reusability, suggesting broad prospects for application due to these exceptional properties.

## Figures and Tables

**Table 1:** Content of C, H, N in microcrystalline cellulose, Cell-g-PBA, Cell-g-P(BA-MBA) and P(BA-MBA).

Sample Name	H (%)	C (%)	N (%)
MCC	6.66	42.23	0
Cell-g-PBA	9.32	62.77	0
Cell-g-P(BA-MBA)	9.25	65.40	<0.1
P(BA-MBA)	9.78	65.56	<0.1

**Table 2:** Comparison of oil-adsorption capacity of several reported adsorbents.

Adsorbents	Oil Adsorption Capacity (g/g)	References
PS-PMMA/DVB	1.5–12.1	[32]
PP/PTFE fibrillar blend	5–24	[33]
Supermacroporous Polystyrene materials	29.5	[34]
Porous Al_2_O_3_/acrylic resin composites	6.8–30.1	[25]
Hollow Fiber MgO Nanoparticle Resin composite	10.4–28.2	[35]
Cell-g-P(BA-MBA)	0.5–37.6	This work

**Table 3:** Related parameters of pseudo-first-order kinetic model and pseudo-second-order kinetic model.

		Pseudo-First-Order			Pseudo-Second-Order		
Oil	q_e,exp_ (g/g)	k_1_ (min^−1^)	q_e,calc_ (g/g)	R^2^	k_2_ (g/g min^−1^)	q_e,calc_ (g/g)	R^2^
Chloroform	37.55	0.0176	32.55	0.98695	0.0021	36.98	0.99353
Toluene	18.72	0.0278	14.53	0.95609	0.0018	17.16	0.9869
Gasoline	10.45	0.0180	10.46	0.98089	0.0016	11.88	0.9882

**Table 4:** Related parameters of Langmuir adsorption isotherm and Freundlich adsorption isotherm.

Oil	Langmuir Isotherm			Freundlich Isotherm		
	q_max_ (g/g)	K_L_ (L/g)	R^2^	K_F_ (g/g)	n	R^2^
gasoline	10.12	0.57	0.94653	3.44	1.78	0.99094

**Table 5:** Thermodynamic parameters for oil adsorption of Cell-g-P(BA-MBA) in gasoline/water system.

T (K)	∆G^0^ (kJ mol^−1^)	∆H^0^ (kJ mol^−1^)	∆S^0^ (J mol^−1^ K^−1^)	R^2^
293.15	−1.3547			
303.15	−1.1151	−9.7099	−0.0285	0.9970
313.15	−0.8162			
323.15	−0.5027			

## Data Availability

Data are contained within the article.
